# Axially Disubstituted Silicon(IV) Phthalocyanine as a Potent Sensitizer for Antimicrobial and Anticancer Photo and Sonodynamic Therapy

**DOI:** 10.3390/ijms26157447

**Published:** 2025-08-01

**Authors:** Marcin Wysocki, Daniel Ziental, Zekeriya Biyiklioglu, Malgorzata Jozkowiak, Jolanta Dlugaszewska, Hanna Piotrowska-Kempisty, Emre Güzel, Lukasz Sobotta

**Affiliations:** 1 Chair and Department of Inorganic and Analytical Chemistry, Poznan University of Medical Sciences, Rokietnicka 3, 60-806 Poznan, Poland; marcin.wysocki@student.ump.edu.pl (M.W.); dziental@ump.edu.pl (D.Z.); 2 Doctoral School, Poznan University of Medical Sciences, Bukowska 70, 60-812 Poznan, Poland; malgorzata.jozkowiak@gmail.com; 3 Department of Chemistry, Faculty of Science, Karadeniz Technical University, Trabzon 61080, Türkiye; 4 Chair and Department of Toxicology, Poznan University of Medical Sciences, Rokietnicka 3, 60-806 Poznan, Poland; hpiotrow@ump.edu.pl; 5 Division of Anatomy, Department of Human Morphology and Embryology, Faculty of Medicine, Wroclaw Medical University, Chalubinskiego 6a, 50-368 Wroclaw, Poland; 6 Chair and Department of Genetics and Pharmaceutical Microbiology, Poznan University of Medical Sciences, Rokietnicka 3, 60-806 Poznan, Poland; jdlugasz@ump.edu.pl; 7 Department of Engineering Fundamental Sciences, Sakarya University of Applied Sciences, Sakarya 54050, Türkiye; eguzel@subu.edu.tr

**Keywords:** phthalocyanine, sensitizer, antimicrobial, photodynamic therapy, sonodynamic, therapy, bacteria

## Abstract

The unique properties of phthalocyanines (Pcs), such as strong absorption, high photostability, effective singlet oxygen generation, low toxicity and biocompatibility, versatile chemical modifications, broad spectrum of antimicrobial activity, and synergistic effects with other treatment modalities, make them a preferred superior sensitizer in the field of antimicrobial photodynamic therapy. The photodynamic and sonodynamic activity of 3-(3-(diethylamino)phenoxy)propanoxy substituted silicon(IV) Pc were evaluated against bacteria and cancer cells. Stability and singlet oxygen generation upon light irradiation and ultrasound (1 MHz, 3 W) were assessed with 1,3-diphenylisobenzofuran. The phthalocyanine revealed high photostability in DMF and DMSO, although the singlet oxygen yields under light irradiation were low. On the other hand, the phthalocyanine revealed excellent sonostability and caused a high rate of DPBF degradation upon excitation by ultrasounds at 1 MHz. The silicon phthalocyanine presented significant bacterial reduction growth, up to 5 log against MRSA and *S. epidermidis* upon light excitation, whereas the sonodynamic effect was negligible. The phthalocyanine revealed high activity in both photodynamic and sonodynamic manner toward hypopharyngeal tumor (FaDu, 95% and 42% reduction, respectively) and squamous cell carcinoma (SCC-25, 96% and 62% reduction, respectively). The sensitizer showed ca. 30% aldehyde dehydrogenase inhibition in various concentrations and up to 85% platelet-activating factor acetylhydrolase for 0.25 μM, while protease-activated protein C was stimulated up to 66% for 0.75 μM.

## 1. Introduction

Phthalocyanines (Pcs) are synthetic macrocyclic compounds whose structures are related to the naturally occurring porphyrins. They are known as potent photosensitizers (PSs) with strong absorption between 600 and 800 nm, in the red-light part of the spectrum [1,2,3,4,5]. As with other porphyrinoids, Pcs are prone to various modifications, the most common of which is the formation of complexes with other elements, usually metals. The incorporation of metals and metalloids often enhances the properties of macrocyclic compounds. The principle of PDT is focused on ROS formation in the target site [3,4,6,7,8,9,10].

The Pcs are widely recognized sensitizers in terms of photodynamic therapy (PDT) and sonodynamic therapy (SDT), two similar techniques that benefit from the interaction of the sensitizer with light or ultrasounds, respectively. Such interactions can initiate further chemical reactions between the excited sensitizer and surrounding molecules, such as molecular oxygen or organic moieties [11]. Despite their similarities, both techniques have different mechanisms. For PDT, the key interaction is photon absorption by the sensitizer following its excitation. Next, the excited sensitizer undergoes deactivation by forming reactive oxygen species (ROS), including singlet oxygen. The drawback of this approach is associated with limited tissue penetration by light (photons), strongly dependent on the wavelength. Phthalocyanines are good candidates for PS in PDT due to strong light absorption within the so-called “phototherapeutic window” range (600–800 nm) while light provides enough energy to the molecule [8,12]. Interestingly, the processes occurring during SDT work in a slightly different manner. The energy is passed to the sensitizer (sonosensitizer, SS) via acoustic cavitation, the phenomenon of creating oscillating microbubbles in response to ultrasound. Irregular oscillation of those bubbles results in their collapse, locally providing extreme conditions that promote the formation of radicals from affected molecules, such as solvent, sensitizer, or other moieties. The radicals may then react with other molecules and generate other radicals, but may also excite the SS. Additionally, the mechanical effects of the ultrasound provide other beneficial effects, such as an increase in cell membrane permeability and mechanical rupture of cellular membranes [13,14,15]. Both processes are characterized by specific chemical reactions that result in the generation of particular ROS, which are potent oxidizers that are utilized to directly oxidize the cellular membranes of targeted cells, either bacterial or cancerous ones [3,13,14,16,17,18,19,20,21].

**Figure 1:**
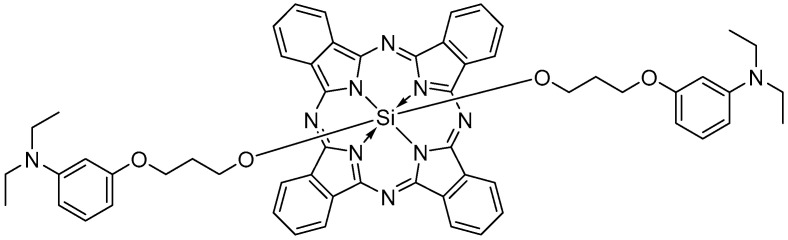
Molecular structure of the studied SiPc.

Interestingly, the unusual coordination of silicon(IV) allows for bearing two additional substituents placed axially to the Pc ring that may effectively improve the compounds’ properties, including solubility and hamper aggregation tendency, due to increased steric hindrance of the macrocycle. The possibility to form the aggregates is one of the key drawbacks in terms of (PDT), due to self-quenching ROS [3,4,7,8]. Axial substituents, characterized by large, branched ligands, reduce the risk of formation of dimers or larger clusters typical of phthalocyanines. Therefore, various silicon(IV) Pcs have been described so far. However, the research on their use in photodynamic and sonodynamic therapy remains very limited, despite versatile synthesis and potentially high therapeutic efficacy. Thanks to analyses conducted by, among others, Chen et al. [22] the introduction of axial substituents in phthalocyanines has been recognized as a promising strategy for modifying their photochemical properties. Axial substitution enhances nonlinear optical absorption by introducing a dipole moment perpendicular to the macrocycle and improving excited-state absorption through stabilization of the excited state. It also allows fine-tuning of spectral characteristics and excited-state lifetimes, contributing to greater control over the photophysical behavior of the molecule [23]. In Si(IV) phthalocyanines, axial ligands can modulate the electron density of the macrocycle through inductive or mesomeric effects without directly perturbing the conjugated system. Electron-donating groups such as diethylamino may induce a slight bathochromic shift in absorption, potentially extending it toward the near-infrared region, which is favorable for applications requiring deeper light penetration, such as photodynamic therapy [24,25]. In biological studies, diethylamino groups are one of the most important compounds thanks to their various biological activities such as anticancer, photodynamic therapy [26,27,28]. For this purpose, (3-(diethylamino)phenoxy) propanoxy groups were preferred as substituents because they contain propanoxy chains for enhanced organic solubility and quaternizable nitrogen atoms. Motivated by these facts, it has been focused on non-aggregated silicon(IV) phthalocyanine as a strategy to explain Pcs in conjunction with the 3-(3-(diethylamino)phenoxy)propanoxy group to study antimicrobial and anticancer photo-sonodynamic therapy. There is no study in the literature in which diethylamino groups substituted with silicon phthalocyanine are used as sensitizers for either photodynamic or sonodynamic activity. This is the first study of axially substituted silicon phthalocyanine containing 3-(3-(diethylamino)phenoxy)propanoxy groups as potential sensitizers for photodynamic and sonodynamic purposes. Thus, we present the photo- and sonochemical properties and activity toward bacterial and cancer cells in vitro of silicon(IV) Pc bearing axial 3-(3-(diethylamino)phenoxy)propanoxy substituents (SiPc, Figure 1). The synthesis and electrochemical properties of the compound have been reported before [29].

## 2. Results and Discussion

**Figure 2:**
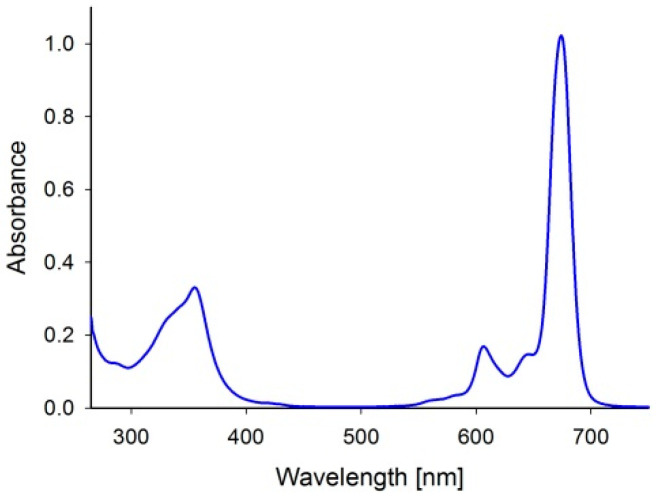
Spectrum of the studied SiPc dissolved in DMF (6.08 × 10^−6^ M).

Evaluated silicon(IV) phthalocyanine bearing axial 3-(3-(diethylamino)phenoxy)propanoxy substituents (SiPc) revealed two absorption bands in the 300–400 nm range—the Soret band and in the 600–700 nm range—the Q band (Figure 2).

These absorption bands are typical for phthalocyanines and the most important from the PDT point of view is the Q band located at the “therapeutic window” region [1]. Following light activation, singlet oxygen quantum yield values were close to zero—see Table 1.

**Figure 3:**
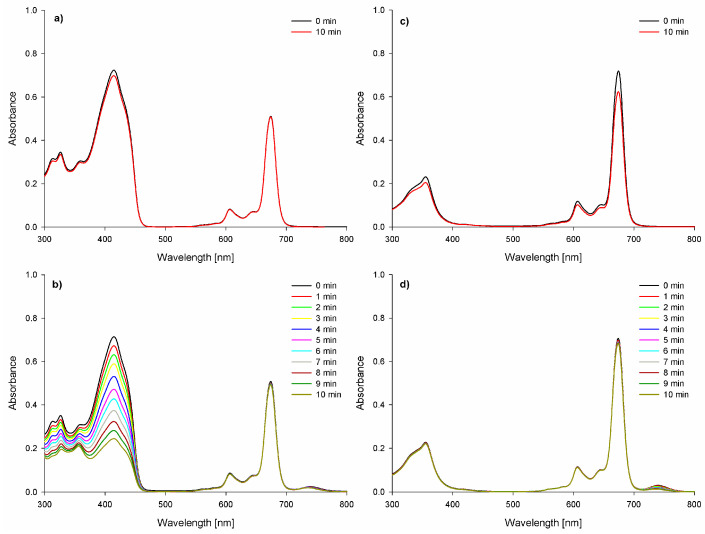
DPBF decomposition in the mixture with SiPc in DMF activated by: (**a**) light (0.5 mW/cm^2^; 660 nm; c_SiPc_ = 2.58 × 10^−6^ M, c_DPBF_ = 3.42 × 10^−5^ M); (**b**) ultrasounds (3 W, 1 MHz, 40% duty cycle); (c_SiPc_ = 2.57 × 10^−6^ M, c_DPBF_ = 3.46 × 10^−5^ M); SiPc decomposition in DMF exposed to: (**c**) light (>450 nm); (**d**) ultrasounds (3 W, 1 MHz, 40% duty cycle).

Interestingly, the rate of DPBF decomposition in the presence of phthalocyanine differed significantly depending on the used trigger—light or ultrasounds (Figure 3). It is well-known that silicon(IV) phthalocyanines can produce singlet oxygen both with very high and low Φ_Δ_ values [34,35,36,37]. SiPc is a very poor singlet oxygen generator under light exposure, which can be associated with axial substituents bearing nitrogen atoms in the aliphatic chain. When confronted with the relatively high biological activity, these results suggest that other molecular factors may play a key role in the photodynamic process. Some previously described silicon phthalocyanines exhibit relatively long excited-state lifetimes, which may compensate for lower ROS generation efficiency [38,39]. Additionally, the positive charge of the molecule may enhance its interactions with the cell membrane or wall, thereby potentiating the phototoxic effect [40]. In comparison with other silicon phthalocyanines exhibiting higher singlet oxygen generation potential, a key distinguishing factor appears to be the presence or absence of strong electron-donating groups, long-chain alkyl or bulky substituents, or halogen atoms such as bromine, chlorine, or fluorine [41].

**Figure 4:**
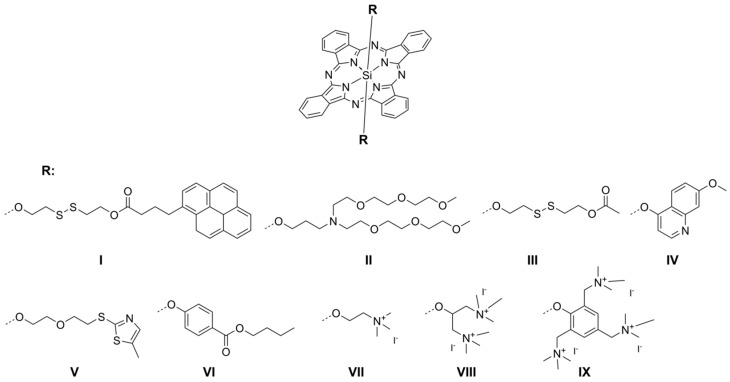
The molecular structures of silicon(IV) derivatives **I**–**IX**.

A similar phenomenon has been reported by van de Winckel et al. [33] for compounds **I**–**III** (Figure 4). Values of Φ_Δ_ dependent on substituent were in the range from ca. 0.03 for **II** to ca. 0.38 for **III** [34,35]. Surprisingly, the nitrogen atom which is included in the aromatic ring does not cause a dramatic drop in singlet oxygen formation. It was reported high Φ_Δ_ values for phthalocyanines **IV** (0.62) and **V** (0.86) [36,42]. Additionally, axial substituents bearing aliphatic chains in their structure without nitrogen atoms present high Φ_Δ_ values, i.e., **VI** (0.48) [35]. On the other hand, the presence of quaternized nitrogen atoms, results in quenching ^1^O_2_ formation as well, i.e., **VII** (0.11), **VIII** (0.11), and **IX** (0.08) (Figure 4) [37].

**Figure 5:**
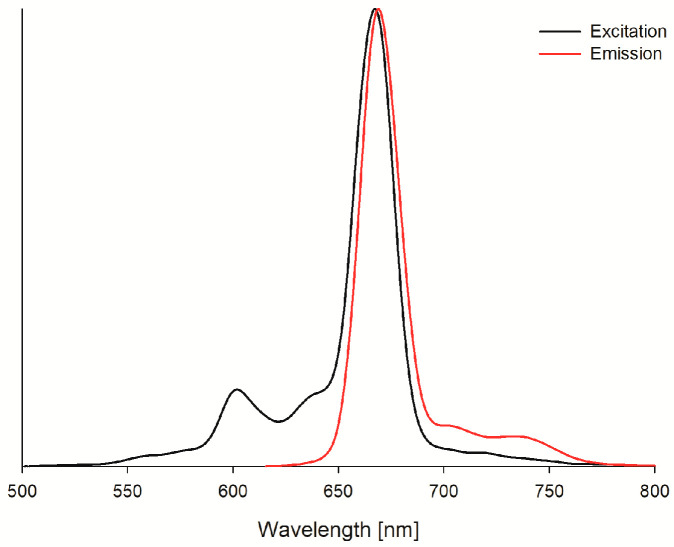
The fluorescence spectra for SiPc in DMF (excitation wavelength λ_ex_ = 667 nm).

The photostability of SiPc was evaluated as well in a liquid environment (DMF and DMSO). SiPc under light exposure decomposed according to the photobleaching mechanism. It was observed that the absorption bands were vanishing, without forming new ones (Figure 3c). The quantum yield values (18.9 × 10^−6^ and 34.1 × 10^−6^ in DMF and DMSO, respectively) of photodegradation of the presented photosensitizer classify it as a stable compound [43]. The ability of SiPc to emit light (Figure 5) has also been evaluated. Its fluorescence quantum yields were determined at the level of 0.07 (Table 1, Figure 5), which are much lower in comparison to the values of the Φ_f_ for the standard.

In the sonochemical experiments, it was noticed that SiPc upon sonication efficiently decomposes DPBF. The half-life time of DPBF dropped over 5-fold in the presence of sensitizer (Table 2). The decomposition DPBF rate for SiPc under sonication is over two times faster in comparison to lately reported porphyrazines [12]. This potentially enables to achievement of high sonodynamic activity in biological evaluation. Notably, the presented compounds demonstrated the highest sonostability (T_0.5_ = 7737.3 min, Figure 3d) reported to date among silicon phthalocyanines studied in sonochemistry [12]. Low frequencies resulted in a decomposition sensitizer. On the other hand, high frequencies >> 1 MHz (i.e., 2 MHz) resulted in no ROS formation.

The antibacterial evaluation indicated that SiPc under light irradiation is extremely active. For activation light at the dose of 100 and 150 J/cm^2^ was used. Surprisingly, PS activation at 100 J/cm^2^ did not induce any activity against MRSA, whereas against *Staphylococcus epidermidis* high—ca. 5 log reduction in bacterial growth was noticed (Table 3). When the light dose is raised to 150 J/cm^2^ the bacterial growth reduction achieves the rate of ca. 5 log for both species. The obtained here results in comparison to those reported by van de Winckel et al. where silicon(IV) phthalocyanines at the concentration of 20 µM (here only 10 µM) were activated with comparable light dose have given maximally ca. 4 log reduction in *S. aureus* growth [34]. In the sonodynamic evaluation, no activity against bacteria was noted.

Similarly, in the SiPc activity evaluation against cancer cells, much higher anticancer activity in the photodynamic than sonodynamic pathway was observed (Table 4). For the experiments, the squamous cell carcinoma (SCC-25) and hypopharyngeal tumor cell line (FaDu) cell lines were chosen. These tumors are one of the most common problems of head and neck oncology. Interestingly, an increase in light dose from 5 to 10 J/cm^2^ did not result in a rise in the activity for all tested cells. On the other hand, when comparing the viability of cancer cells treated with PDT-SiPc (viability reduction about >94%) to the data obtained before, for similar silicon(IV) phthalocyanine (10 µM), viability reduction was about ca. 60% [44]. Unfortunately, the highest activity of the tested sensitizer was shown against MRC-5 fibroblasts. This indicated that in the next stage of evaluation, application, and excitation with light of the sensitizer have to be performed only within the diseased area. In contrast, to what was reported before for sonodynamically inactive porphyrazine/phthalocyanine hybrids against cancer cells, studied here, SiPc shows moderate activity with cell viability reduction 41.5–82.5% (Table 4).

**Figure 6:**
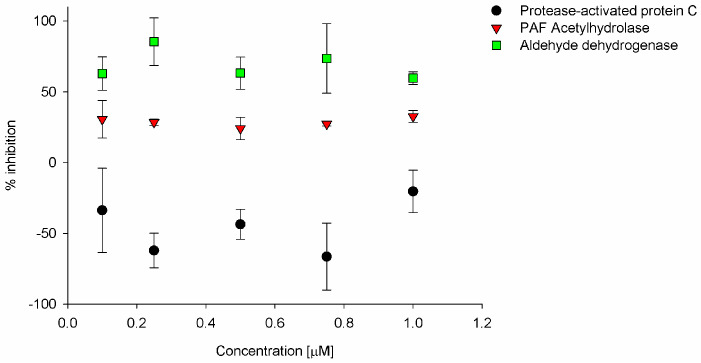
Enzymatic inhibition activity of SiPc (*p* < 0.05).

The impact of SiPc on the activity of enzymes was also evaluated. Highly inhibited was aldehyde dehydrogenase at about 85% in the sensitizer concentration of 0.75 µM (Figure 6). Such a rate of inhibition may indicate additional independent of the studied compound’s light mechanism of anticancer activity. Otherwise, tested phthalocyanine reduced the activity of PAF acetylhydrolase at ca. 40% independently of the concentration within the studied range. Interestingly, our sensitizer increased the activity of protease-activated protein C, which is responsible for anticoagulant and anti-inflammatory activity [45]. Additional stimulation of protease-activated protein C may limit inflammation associated with the PDT procedure. Nevertheless, promising results of the impact of SiPc on enzymatic activity have to be further evaluated.

The obtained results represent an interesting alternative to currently available treatment methods, particularly for surface infections. The development of optimal sonosensitizers will enable further combination with carriers, such as hydrogel patches propagating ultrasound, as innovative solutions for treating diabetic foot ulcers and hard-to-heal, superinfected wounds. This technology appears particularly promising for multidrug-resistant and mixed infections, where selecting the optimal antibiotic therapy can be especially challenging. Moreover, in cancer treatment, this method could serve as a valuable adjuvant therapy with a relatively low risk of severe adverse effects and side effects.

## 3. Materials and Methods

### 3.1. Materials

Dimethyl sulfoxide (DMSO, 99.9% for spectroscopy) and N,N-dimethylformamide (DMF, ≥99.8% for spectroscopy ACS) were purchased from Fisher (Waltham, MA, USA). Zinc(II) phthalocyanine (ZnPc) and 1,3-diphenylisobenzofuran (DPBF) were purchased from Aldrich (St. Louis, MO, USA). Brain-heart infusion (BHI) broth was purchased from Oxoid (Waltham, MA, USA). The SCC-25 and FaDu human squamous cell carcinoma cell lines and the MRC-5 human diploid lung fibroblasts were purchased from the American Type Culture Collection (ATCC) (Manassas, VA, USA). Phosphate-buffered saline, Dulbecco’s Modified Eagle’s Medium/F-12 (DMEM/F-12), Fetal Bovine Serum (FBS), L-glutamine, Penicillin, Streptomycin, Eagle’s Minimum Essential Medium (EMEM), Trypsin-EDTA solution, Dimethyl sulfoxide (DMSO, for cell culture), and MTT were purchased from Merck (Rahway, NJ, USA). Purified acetylcholinesterase (C3389), acetylcholinesterase inhibitor screening kit (MAK324), purified aldehyde dehydrogenase (A6338), aldehyde dehydrogenase inhibitor screening kit (MAK327), and activated protein C (APC) inhibitor screening kit (MAK346) were purchased from Merck. A platelet-activating factor acetylhydrolase (PAF-AH) inhibitor screening kit was purchased from BioVision (Exton, PA, USA).

### 3.2. Photochemistry Measurements

#### 3.2.1. Singlet Oxygen Quantum Yields Determination Under Light Exposure

The measurements of singlet oxygen quantum yields were conducted in DMSO and DMF at ambient temperature, according to the comparative method described previously [12,46,47,48]. The method involves using DPBF as a chemical quencher of singlet oxygen and ZnPc as a reference compound. Firstly, irradiation of the respective sensitizer-quencher mixtures with light of fixed power (0.5 mW/cm^2^ measured with RD 0.2/2 TD probe radiometer, Optel, Opole, Poland) was performed, and the proper wavelength of the Q band region (660 nm) was determined. Then, spectral changes allowed calculation of the quencher (DPBF), and decomposition kinetics were recorded. Such data obtained for mixtures of DPBF with ZnPc (reference) and mixtures of DBPF with the studied compound (SiPc) were then compared according to Equation (1):

(1) $$\Phi_{\Delta }=\Phi_{\Delta }^{std}\frac{RI_{Abs}^{Std}}{R^{Std}I_{Abs}},$$

where $\Phi_{\Delta }^{std}$ is the quantum yield of singlet oxygen known for ZnPc (0.56 in DMF and 0.67 in DMSO), *R* and *R^Std^* stand for rates of DPBF decomposition, and *I_Abs_* with $I_{abs}^{Std}$ stand for rates of light absorption of both investigated SiPc and the standard, respectively. The light emitted by 150 W xenon lamp (Optel, Opole, Poland) was adjusted to the proper wavelength with the monochromator (M250/1200/U with 2 nm/mm (Dk = 4 nm) dispersionOptel, Opole, Poland). The absorption spectra and their changes (within 10 min of the experiment provided energy of 300 mJ/cm^2^) were gathered using an OceanOptics Flame spectrophotometer coupled with DT-MINI-2-GS light source. The obtained data were then used for calculations of singlet oxygen quantum yields using the presented equation [12,31,46,47,48].

#### 3.2.2. Photodecomposition Quantum Yields Determination

The measurements of photostability were performed in DMF and DMSO. The samples were prepared under aerobic conditions and kept at ambient temperature and the investigation was conducted according to the method described before [12,46,48]. As a light source served the 150 W xenon lamp (Optel, Opole, Poland), and the visible light (>450 nm; 20 mW/cm^2^) was filtered with HCC-16 cutting glass filter. Absorption spectra changes during the irradiation (10 min provided total energy of 12 J/cm^2^) were recorded with OceanOptics Flame spectrophotometer equipped with DT-MINI-2-GS light source, and the photodegradation quantum yield was then calculated according to the equation presented before [12].

#### 3.2.3. Fluorescence Measurements

The fluorescence spectra were recorded in DMF and DMSO at 21 °C using Jasco FP-6200 spectrofluorometer with the slit width set to match 5 nm bandwidth. Before measurements, the absorbance values of the samples in the excitation maximum were set to 0.1 and placed in quartz cuvettes (l = 10 mm), and then the excitation and emission spectra were recorded. For calculations of the fluorescence quantum yields, ZnPc was employed as a reference compound. The fluorescence quantum yields were then calculated using Equation (2):

(2) $$\Phi_{f}=\Phi_{f}^{std}\frac{FA_{Ex}^{std}}{F^{std}A_{Ex}},$$

where $\Phi_{f}^{std}$—fluorescence quantum yield for ZnPc (0.30 in DMF and 0.20 in DMSO), *F* and *F^Std^*—fluorescence intensity described as the spectrum integral for the sample and the standard, *A_Ex_* and $A_{Ex}^{std}$—absorbance of the samples at the excitation wavelength (for SiPc 667 nm in DMF, 669 nm in DMSO) [30,33].

### 3.3. Sonochemistry Measurements

#### 3.3.1. DPBF Decomposition Under Sonication

The SiPc-mediated decomposition of DPBF was measured in the dark and at ambient temperature. The mixtures of the phthalocyanine with DPBF were aerated for 10 min, then moved into quartz cuvettes (l = 10 mm), and sealed with plugs. Before the measurements, each cuvette was shaken and the spectrum before sonication was recorded using a Shimadzu U-1900 spectrophotometer (Shimadzu, Tokyo, Japan). The cuvettes were then thoroughly shaken again and placed in the ultrasonic apparatus (constructed by the Institute of Fundamental Technological Research, Polish Academy of Sciences, Warsaw, Poland). The ultrasounds of 1 MHz frequency 3 W power and 400% duty cycle were transmitted via an ultrasonic head for ten 1 min intervals. The absorption spectra were recorded after each interval and the samples were subsequently shaken before further sonication. For the calculations, mean values of the data obtained from five independent measurements were taken.

#### 3.3.2. Sonostability of Sensitizers

The sonostability measurements of SiPc were conducted according to the same steps described in Section 3.3.1.

### 3.4. Antibacterial Activity

#### 3.4.1. Cell Culture

The methicillin-resistant *Staphylococcus aureus* (MRSA, clinical strain) and *Staphylococcus epidermidis* (clinical strain) strains were cultured aerobically in brain heart infusion (BHI) broth at 36 ± 1 °C for 24 h. The microbes were then centrifuged at 3000 rpm for 15 min, harvested, and resuspended in 10 mM phosphate-buffered saline (pH = 7.0). The cells were later diluted to approximately 10^7^ colony-forming units per ml (CFU/mL). The bacterial suspension was placed into wells of a 96-well plate, and then aliquots of a proper amount from the stock solution prepared in DMSO to obtain the final concentration of 10 µM were added. Control cells were cultured under the same conditions with 0.1% DMSO. The reduction in bacterial growth was calculated using the colony counting method following the guidelines of CLSI. Experiments were performed in triplicate.

#### 3.4.2. Photodynamic Pathway

The plate was placed in the dark for 30 min to incubate properly, then subjected to irradiation with 660 nm light using a custom LED lamp (20 mW/cm^2^ Led-Byt, Bytom, Poland), achieving the dose of 100 (80 min) and 150 J/cm^2^ (120 min). The dose and irradiation time were evaluated with RD 0.2/2 radiometer (Optel, Opole, Poland). Control samples without the sensitizer presence were performed as well. After the given irradiation interval, the bacterial suspensions were diluted, placed on tryptic soy agar plates and incubated for 24 h at 36 ± 1 °C.

#### 3.4.3. Sonodynamic Pathway

The incubation was performed via placing the plate in dark place for 30 min. After incubation, the plate was positioned above the ultrasonic head (the Institute of Fundamental Technological Research, Polish Academy of Sciences, Warsaw, Poland) and subjected to sonication (1 MHz, 3 W, 40% duty cycle), reaching a final dose of 1024 J/cm^2^. Control samples, not including the sensitizer and ultrasound, were also incorporated. The bacterial cultures after the sonication were moved to tryptic soy agar plates and incubated at 36 ± 1 °C for 24 h.

### 3.5. Anticancer Activity

#### 3.5.1. Cell Culture and Viability Assay

The chosen cell lines: human squamous cell carcinoma SCC-25 and FaDu, as well as the reference human diploid lung fibroblasts MRC-5 were cultivated in the optimal conditions (37 °C, 95% humidity, 5% CO_2_ content) using an incubator. FaDu and MRC-5 cell lines were cultivated in phenol red-free Eagle’s Minimum Essential Medium (EMEM) supplemented with 10% fetal bovine serum (FBS), 2.5 mM L-glutamine, penicillin (100 U/mL), and streptomycin (0.1 mg/mL), while the SCC-25 cell line was cultured in phenol red-free Dulbecco’s Modified Eagle Medium F-12 (DMEM/F-12) medium complemented with 10% FBS, 2.5 mM L-glutamine, penicillin (100 U/mL), and streptomycin (0.1 mg/mL). The cell cultures were harvested using trypsin-EDTA solution and seeded in 6-well cell culture plates at 10^6^ cells per well. The cells were left overnight to attach and then the stock solution of the silicon phthalocyanine in DMSO was added to maintain the final concentration of 10 μM. Control cells were cultured in the same conditions with 0.1% DMSO instead. Cell viability was assessed with an MTT test the next day after treatment. The culture medium was replaced with mixtures of MTT and EMEM or DMEM/F-12 (both 1:8), depending on the investigated cell lines. The resulting formazan crystals were then dissolved by adding 1 mL of DMSO, and the absorbance was measured using an Elx-800 plate reader (BioTek, Bad Friedrichshall, Germany) at 570 nm (reference wavelength 650 nm). Experiments were performed in triplicate.

#### 3.5.2. Photodynamic Pathway

The cells were incubated for 5 h and then exposed to 660 nm LED light (Led-Byt, Bytom, Poland) to achieve the final light doses of 5 and 10 J/cm^2^. The cell viability was then measured using an MTT assay.

#### 3.5.3. Sonodynamic Pathway

After 5 h of incubation, the cells were exposed to ultrasound using a transducer with a resonance frequency of 3 MHz (continuous mode) until the energy dose reached 96 J/cm^2^. Then, the cell viability was assessed with an MTT assay.

### 3.6. The Impact of Sensitizer Activity on Endogenous Enzymes

The assays were performed according to the procedures provided by suppliers. The evaluated macrocycle was added in the concentration range of 0.1–1.0 µM. Absorbance was recorded with Tecan Infinite 200 Pro MNano (Tecan Life Sciences, Zürich, Switzerland), equipped with an absorbance–monochromator module.

## 4. Conclusions

In a nutshell, silicon(IV) phthalocyanine bearing axial 3-(3-(diethylamino)phenoxy)propanoxy substituents (SiPc) was evaluated, and when excited, this sensitizer decomposed DPBF, and the rate of DPBF bleaching differed significantly depending on the used trigger—light or ultrasounds. The studied SiPc under light exposure decomposed according to the photobleaching mechanism with a low quantum yield. Upon sonication, the efficiently studied sensitizer decomposes DPBF. This potentially enables to achievement of high sonodynamic activity in biological tests. The antibacterial evaluation indicated that SiPc studied here under light irradiation is extremely active. In the sonodynamic triggering of ROS formation by SiPc, no activity against bacteria was noted. Similarly, in the SiPc activity evaluation against cancer cells, much higher anticancer activity in the photodynamic than sonodynamic pathway was observed. The impact of SiPc on the activity of enzymes was also evaluated. Highly inhibited was aldehyde dehydrogenase, about 85%; such a rate of inhibition could indicate additional independence of the light mechanism of the anticancer activity of the studied compound. Interestingly, as presented here, the sensitizer increased the activity of protease-activated protein C. Eventually, SiPc shows promise as a potential sonophotodynamic agent in antimicrobial PDT applications.

## Figures and Tables

**Table 1:** Quantum yields of fluorescence, photodecomposition and singlet oxygen formation (SiPc—studied sensitizer; ZnPc—unsubstituted zinc(II) phthalocyanine; Φ_f_—quantum yield of fluorescence; Φ_P_—quantum yield of photodecomposition; Φ_Δ_—quantum yield of singlet oxygen formation).

Compound	Solvent	Φ_f_	10^6^ Φ_P_	Φ_Δ_
SiPc	DMF	0.07	18.9	0.01
	DMSO	0.07	34.1	0.09
ZnPc	DMF	0.30 [30]	10.2 [31]	0.56 [32]
	DMSO	0.20 [33]	3.5 [31]	0.67 [32]

**Table 2:** Kinetic parameters of: sonosensitizer decomposition upon sonication; DPBF decomposition by sensitizer excited with ultrasounds [1 MHz] (brackets denote the parameters of sensitizer decomposition under ROS formation in the experiment with DPBF).

	DPBF	SiPc	DPBF in the Presence of SiPc
k (min^−1^)	0.0245	0.0000896	0.134 [0.00081]
t_0.5_ (min)	28.3	7737.3	5.2 [858.2]
Ln (A)	−0.0165	0.009	−0.086 [0.011]

**Table 3:** Antibacterial activity in photodynamic and sonodynamic pathway (n.a.—no activity, * for *p* < 0.05).

Log Reduction in Bacterial Growth				
photodynamic activity			positive control	
Light dose [J/cm^2^]	MRSA	*S. epidermidis*	Light at 660 nm	SiPc in the dark
100	n.a.	4.96 ± 0.53 *	n.a.	
150	5.25 ± 0.36 *	4.96 ± 0.47 *		
sonodynamic activity			positive control	
Ultrasound dose [J/cm^2^]	MRSA	*S. epidermidis*	Ultrasound	SiPc in the dark without ultrasound
512	n.a.	n.a.	n.a.	
1024				

**Table 4:** Anticancer activity via photodynamic and sonodynamic pathway. It is shown the percentage of viable cells. (sensitizer concentration—10 µM is a table).

Cell Line	Control	Positive Control				Photodynamic Therapy		Sonodynamic Therapy
		SiPc	Light at 660 nm [5 J/cm^2^]	Light at 660 nm [10 J/cm^2^]	US	SiPc Activated with 660 nm Light		SiPc Activated with US [3 W, 1 MHz, 40% Duty Cycle]
						5 [J/cm^2^]	10 [J/cm^2^]	
viability [%]								
MRC-5	100.00 ± 6.87	63.20 ± 3.92 *	67.41 ± 0.87 *	67.55 ± 2.51 *	93.51 ± 7.10	1.27 ± 0.05 *	1.2 ± 0.05 *	18.5 ± 1.88 *
SCC-25	100.00 ± 2.98	78.89 ± 0.79 *	85.89 ± 1.48 *	92.95 ± 1.39	94.08 ± 4.88	3.56 ± 0.07 *	5.79 ± 0.95 *	37.45 ± 14.28 *
FaDu	100.00 ± 3.04	94.75 ± 4.29	65.86 ± 5.28 *	57.54 ± 2.91 *	77.64 ± 13.36 *	5.99 ± 0.44 *	4.72 ± 0.05 *	58.53 ± 14.03 *

* for *p* < 0.05.

## Data Availability

All data are included in the manuscript or the Supplementary Materials. Additional details will be provided upon request.

## Supplementary Materials

The following supporting information can be downloaded at: https://www.mdpi.com/article/10.3390/ijms26157447/s1.

## Abbreviations

The following abbreviations are used in this manuscript:

PS	Photosensitizer
PDT	Photodynamic therapy
ROS	Reactive oxygen species
SS	Sonosensitizer
SDT	Sonodynamic therapy
Pc	Phthalocyanine
DPBF	1,3-diphenylisobenzofuran
DMSO	Dimethyl sulfoxide
DMF	N,N-dimethylformamide
ZnPc	Unsubstituted zinc(II) phthalocyanine
CFU	Colony-forming units
SiPc	Evaluated here silicon(IV) phthalocyanine
MRSA	Methicillin-resistant *Staphylococcus aureus*
